# Clinical effectiveness of a child-specific dynamic stretching programme, compared to usual care, for ambulant children with spastic cerebral palsy (SPELL trial): a parallel group randomized controlled trial

**DOI:** 10.1302/2633-1462.65.BJO-2024-0267

**Published:** 2025-05-01

**Authors:** Tim Theologis, Daniel C. Perry, Ines Rombach, David J. Keene, Ioana R. Marian, Morag Andrew, Catherine Barry, Loretta Davis, Gregory Firth, Heidi Fletcher, Beth Fordham, Vivi Gregory Osborne, Helen Gregory Osborne, Lesley Katchburian, Joanna O'Mahoney, Jeremy R. Parr, Rachel Rapson, Jennifer Ryan, Fema Er, Megan Stone, Helen Wood, Sally Hopewell

**Affiliations:** 1 Nuffield Department of Orthopaedics, Rheumatology and Musculoskeletal Sciences University of Oxford, Oxford, UK; 2 University of Liverpool, Liverpool, UK; 3 School of Medicine and Population Health, University of Sheffield, Sheffield, UK; 4 University of Exeter, Exeter, UK; 5 Oxford Clinical Trials Research Unit, Nuffield Department of Orthopaedics, Rheumatology and Musculoskeletal Sciences, University of Oxford, Oxford, UK; 6 Newcastle upon Tyne NHS Foundation Trust; Newcastle University Population Health Sciences Institute, Newcastle upon Tyne, UK; 7 Surgical Interventions Trials Unit, Nuffield Department of Orthopaedics, Rheumatology and Musculoskeletal Sciences, University of Oxford, Oxford, UK; 8 Maidstone and Tunbridge Wells NHS Trust in Kent, Maidstone, UK; 9 Patient and Public Involvement Representative, London, UK; 10 Great Ormond Street Hospital for Children NHS Foundation Trust, London, UK; 11 Children and Family Health Devon, Torbay and South Devon NHS Foundation Trust, Torquay, UK; 12 CP-Life Research Centre, RCSI University of Medicine and Health Sciences, Dublin, Ireland

**Keywords:** Cerebral palsy, Dynamic stretching, Physiotherapy trial, randomized controlled trial, Physiotherapists, physiotherapy, exercise programme, spastic cerebral palsy, Gross Motor Function Classification System, muscle stretching exercises, clinicians, orthopaedic surgery

## Abstract

**Aims:**

Dynamic muscle stretching exercises are one of the interventions frequently prescribed by physiotherapists for children with cerebral palsy (CP). However, there is wide variability in the exercise regimes used and limited evidence of their effectiveness. The SPELL trial will assess the clinical effectiveness of an individually tailored dynamic stretching programme, compared to usual care for ambulant children with spastic CP.

**Methods:**

We are conducting a multicentre, two-arm, parallel group, superiority randomized controlled trial. We will recruit children aged four to 11 years with a diagnosis of spastic CP (bilateral or unilateral) and Gross Motor Function Classification System (GMFCS) levels I to III who are able to comply with assessment procedures and exercise programme with or without support. Participants will be recruited from at least 12 UK NHS Trust physiotherapy and related services. Participants (n = 334) will be randomized (centralized computer-generated one:one allocation ratio) to either: 1) a dynamic stretching exercise programme, with six one-to-one physiotherapy sessions over 16 weeks; or 2) usual NHS care, with a single physiotherapy session and an assessment, and advice regarding self-management and exercise.

**Conclusion:**

The primary outcome is functional mobility measured using the child-/parent-reported Gait Outcomes Assessment List (GOAL) at six months. Secondary outcomes are: joint range of motion (Cerebral Palsy Integrated Pathway protocol) and motor function (timed up and go test) at six months; functional mobility (GOAL) at 12 months; independence (GOAL subdomain A); balance (GOAL subdomain A, B, D); pain and discomfort (GOAL subdomain C); health-related quality of life (youth version of the EuroQol five-dimension questionnaire (EQ-5D-Y)); educational attendance; exercise adherence; and additional physiotherapy treatment at six and 12 months. The primary analysis will be intention to treat.

Cite this article: *Bone Jt Open* 2025;6(5):506–516.

## Introduction

Cerebral palsy (CP) encompasses a group of permanent developmental disorders affecting movement and posture and causing activity limitation.^1^ CP affects approximately one in 400 children in the UK,^2^ and represents a lifetime disability with significant socioeconomic consequences. Functional mobility is best classified by the Gross Motor Function Classification System (GMFCS),^3^ an international standard based on the severity of the motor disability. About 65% children with CP are ambulant, either with walking aids (GMFCS level III) or without (GMFCS levels I and II). CP is also classified according to the affected body areas (one side of the body (unilateral), both sides of the body (bilateral)), and the neurological pattern (spastic, dyskinetic, ataxic, mixed).^1^

In 70% of people, CP predominantly causes spasticity (increased muscle stretch reflex activity and passive stiffness). The increased muscle tone leads to progressive muscle stiffness and deficient longitudinal muscle growth.^4^ This then causes secondary joint contracture, bone deformity, and pain.^5^ Prevention of musculoskeletal deformity and enhancement of motor development are therefore important aims in the management of spastic CP, in order to promote activity and participation.

Physiotherapy is introduced early in the treatment of children and young people with CP to support motor development and prevent musculoskeletal problems.^6^ Physiotherapy provision throughout childhood represents a significant time and cost burden for the child, family, and NHS. The optimization of physiotherapy provision for children and young people with CP was identified as a top priority in the British Academy of Childhood Disability (BACD) James Lind Alliance Childhood Disability Priorities Setting Partnership.^7^ Research on the effectiveness of physiotherapy in preventing deformity and the need for surgery was also prioritized by the British Society for Children’s Orthopaedic Surgery James Lind Alliance Paediatric Orthopaedic Surgery Priorities Setting Partnership.^8^ A recent scoping review also highlighted the need for evidence-based physiotherapy interventions in children and young people with CP, which are deliverable through the NHS and focus on improving activity and participation in a child- and family-friendly manner.^9^

Exercises to stretch tight muscles in order to maintain or improve joint range of motion (ROM) usually form part of physiotherapy treatment.^10^ Stretching can be either static (e.g. manual passive stretching, casting, or splinting) or dynamic, i.e. the individual stretching a muscle by moving the adjacent joints through a full ROM without necessarily holding it to the maximum stretch position. There is wide variation in the physiotherapy techniques used in this field and little evidence supporting any of the stretching regimes.^11,12^ Given the resources, time, and effort required to deliver stretching regimes, there is a pressing need to develop evidence-based practices. A survey of current practice in the UK showed that stretching exercises are one of the interventions frequently used by physiotherapists for children with CP,^13^ with 76% of physiotherapists recommending it frequently and 98% having recommended it at least once in the past year. However, there is wide variability in the stretching regimes used,^14^ limited evidence to support their effectiveness,^11,12^ and protocols are variable.

Dynamic stretching may have advantages over static/passive stretching. Dynamic stretching protocols (actively moving a joint through full ROM without necessarily holding the stretch position) have been widely described in sports science for children and youths who are not affected by neurological conditions. A review of the literature on dynamic stretching noted variation of protocols and the need for consistency in the definition of duration, frequency, number of repetitions, amplitude, velocity, and position.^15^ Detailed descriptions of dynamic stretching protocols exist for typically developing children and young people, but not for those with CP or other neurodisability.^16^ The only trial evaluating a dynamic stretching intervention (backwards downhill walking on a treadmill) showed improved ankle dorsiflexion during walking after the intervention.^17^ Dynamic stretching in children with CP using a six-week robotic programme has been shown to be effective in improving knee motion during gait.^18^ However, this would require extensive resources and would not be feasible and realistic in national health services, such as the UK NHS. Mixed dynamic/passive stretching as part of a wider home-based intervention, also including strengthening, have also been suggested.^19,20^ These paradigms and regimes are mixed, but all proposed similar length of intervention (16 weeks) and frequency (three sessions per week). The pathophysiological and biomechanical advantages of dynamic stretching over passive have also been highlighted: passive stretching lengthens the tendon while dynamic stretching (eccentric muscle contracture) appears to lengthen the muscle.^21-23^

None of the previous studies on dynamic muscle stretching have included a behaviour change component. A dynamic stretching intervention can only be effective if the target population performs and maintains the proposed exercise behaviours. There is evidence to suggest that the addition of behaviour change components to exercise interventions increases the likelihood that the target population will perform the prescribed exercises.^24^ The capability-opportunity-motivation model of behaviour change provides a theoretically based framework for designing complex interventions to enhance behaviour change.^25^

Given the resources, time, and effort for young people, parents, and professionals, required to deliver strengthening regimes, there is a pressing need to evaluate clinical effectiveness.^7,9^ The literature supports testing a clearly defined dynamic stretching intervention that is acceptable to children and families, widely supported by physiotherapists and deliverable in the NHS. As highlighted by the UK’s National Institute for Health and Care Excellence (NICE) guidance on management of spasticity in young people,^26^ the intervention should be child-centred and focused on activity and participation goals.^9^ The burden on the child and family should be minimized and delivery of the intervention should be as unobtrusive as possible.

We describe a randomized controlled trial, using a parallel group design, to assess the effectiveness of an individually tailored dynamic stretching programme, underpinned by evidence-based behaviour change theory, compared to usual care among ambulant children with spastic CP. The dynamic stretching intervention has been co-designed with children and young people with CP, their parents, and healthcare professionals to maximize the deliverability in the context of routine care.

The primary objective is to determine whether an individually tailored 16-week dynamic stretching programme improves functional mobility at six months (measured using the child-/parent-reported Gait Outcomes Assessment List (GOAL) questionnaire),^27^ compared to usual care, among ambulant children with spastic CP.

Secondary objectives are to investigate whether there is any difference between the two groups: at six months, in clinician assessed lower limb joint ROM (Cerebral Palsy Integrated Pathway (CPIP) protocol)^28^ and motor function (timed up and go test);^29^ at 12 months, in functional mobility (GOAL questionnaire);^27^ and at six and 12 months, in independence (GOAL subdomain A), balance (GOAL subdomain A, B, D), pain, and discomfort (GOAL subdomain C), health-related quality of life (youth version of the EuroQol five-dimension questionnaire, EQ-5D-Y),^30^ educational attendance, exercise adherence, and additional physiotherapy treatment.

## Methods

### Study design

**Fig. 1:**
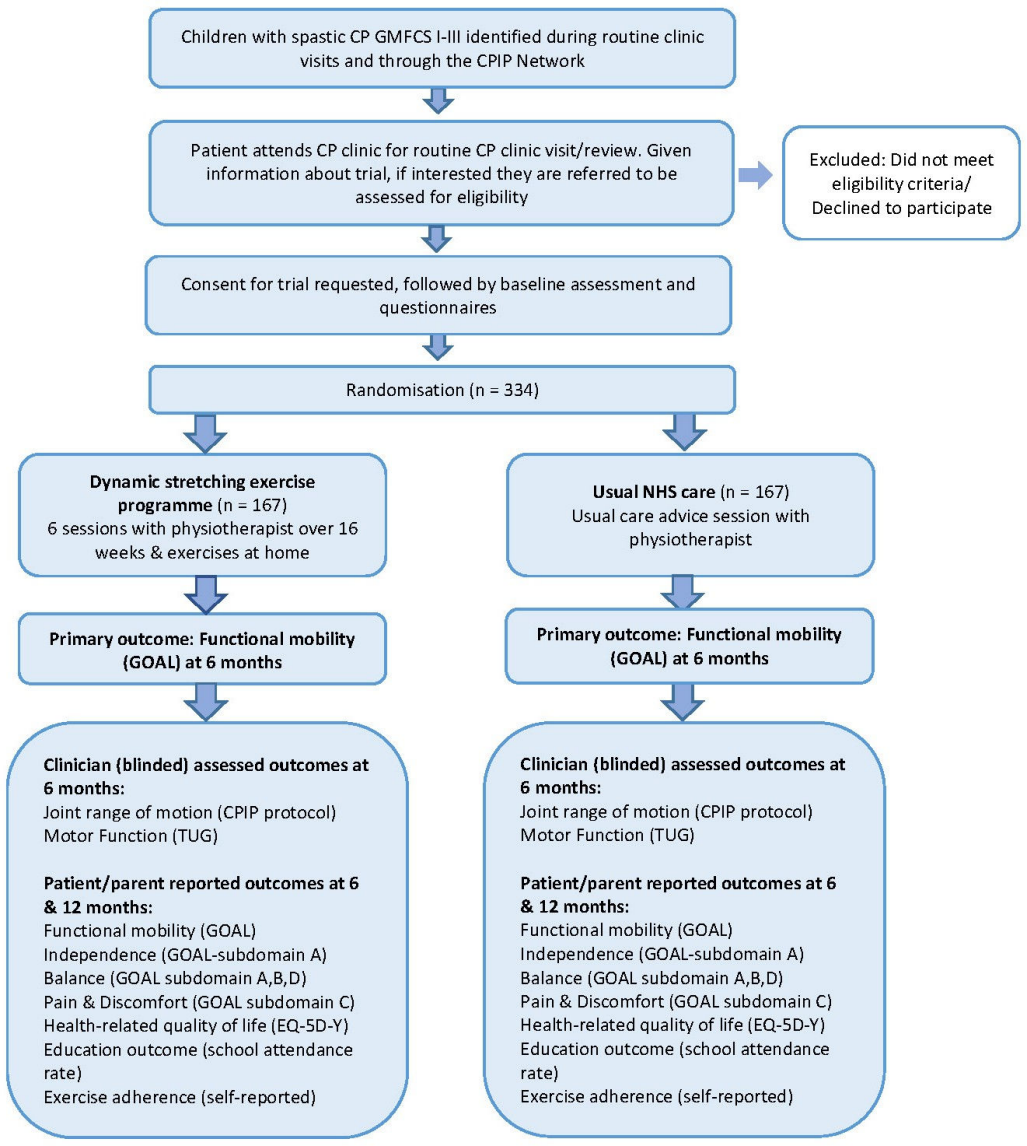
Study flow diagram for SPELL trial. CP, cerebral palsy; CPIP, Cerebral Palsy Integrated Pathway; GOAL, Gait Outcomes Assessment List; EQ-5D-Y, youth version of the EuroQol five-dimension questionnaire; TUG, Timed Up and Go test.

The SPELL trial is a multicentre, two-arm, parallel group, superiority, randomized controlled trial with an embedded internal pilot (Figure 1). SPELL will be conducted alongside the ROBUST trial, which will assess whether an adolescent-specific progressive resistance exercise programme, overseen by a physiotherapist over 16 weeks, improves functional mobility (measured using the child-/parent-reported GOAL questionnaire) at six months in ambulant adolescents aged 12 to 18 years, with spastic CP compared to usual care. The ROBUST trial protocol is published separately.^31^

### Setting

Participants will be identified and recruited from at least 12 UK NHS Trusts, providing paediatric physiotherapy services to children with CP. Sites will be hospital- or community-based, depending on the configuration of local services.

### Study participants

The SPELL trial will recruit children aged four to 11 years (i.e. from their fourth to the day before their 12th birthday) with a diagnosis of predominantly spastic CP (bilateral or unilateral), graded as GMFCS levels I, II, or III. Participants should be able to complete the assessment procedures and the exercise programme, with or without support from their carer, and not regularly be performing a structured exercise programme focused on dynamic stretching as part of their usual physiotherapy routine.

*Eligibility:*

Patients will be eligible for this study if they are:

- aged four to 11 years (i.e. from their fourth to the day before their 12th birthday)
- have a diagnosis of spastic CP (bilateral or unilateral) in GMFCS levels I, II, or III
- willing for their community physiotherapy service and GP to be informed of their participation in the trial.

Patients will be excluded from participation in this study if:

- they had orthopaedic surgery of the lower limbs or selective dorsal rhizotomy in the past 12 months or planned (i.e. date confirmed) in the next six months
- they had lower limb botulinum toxin injections or serial casting in the past four months or planned (i.e. date confirmed) in the next six months
- they are regularly performing a structured exercise programme focused on dynamic stretching as part of their usual physiotherapy routine
- they are unable to comply with the assessment procedures and exercise programme with or without support.

### Recruitment of participants, screening, and eligibility assessment

Children with a diagnosis of spastic CP (GMFCS levels I to III) and who meet current indications for physiotherapy will be screened for eligibility. Sites will identify potential participants through the Cerebral Palsy Integrated Pathway (CPIP) database.^28^ The CPIP database is a national database for the assessment of the musculoskeletal system, including hip surveillance, in children and adolescents with CP. All children with CP are offered an annual CPIP musculoskeletal assessment by a community physiotherapist. Not all people with CP attend hospital; therefore, CPIP offers a unique opportunity to identify children with CP in the community, particularly in underserved areas where access to hospital-based services may be challenging. Not all hospitals are part of the national CPIP database, so potential participants will also be identified and recruited through routine paediatric, orthopaedic, and physiotherapy healthcare visits.

If eligible, children and their parents will be provided information about the trial, including an ‘explainer video’, age-appropriate participant information sheet (i.e. four to seven-year-olds, eight to 11-year-olds parent/guardian participant information sheet), a verbal explanation of the trial and trial procedures, and asked if they wish to be considered for the trial. Those meeting the eligibility criteria and wishing to participate in the trial will then be approached for informed consent. Participants who do not meet the eligibility criteria, or who do not wish to participate, will continue to receive their standard NHS physiotherapy treatment. We will record anonymous information, via site screening logs, on the age, sex, ethnicity, and social deprivation index of those who decline to participate so that we can assess the generalizability of those recruited. The reasons for any potential participants declining to be part of the study will also be recorded.

### Informed consent and baseline assessment

After participants have been assessed for eligibility, informed consent for participation in the trial will be sought by a physiotherapist or other healthcare professional trained in good clinical practice. They will explain the details of the trial and ensure that the potential participant and their parent/guardian has sufficient time to consider participating. Informed consent will be obtained in line with NHS Health Research Authority guidance for research involving children.^32^ Informed consent will usually be obtained electronically in clinic with the consent/assent form being completed directly on the trial database (REDCap).^33,34^ Where appropriate, e-consent may also be obtained remotely, following an initial contact by a member of the site trial team to introduce the study to the participant and their parent/guardian.

Participants, with the support of their parent/guardian, will then be asked to complete the baseline assessment questionnaire that will record simple demographic information and baseline measurements for the primary and secondary outcomes (Table I and Table II). Clinician-assessed outcomes (i.e. lower limb joint ROM and motor function) at baseline will be recorded electronically by a physiotherapist on site.

**Table I.** Timepoints at which outcomes will be assessed.

Outcome	Measurement	Timepoints
**Primary outcome**		
Functional mobility	GOAL questionnaire^27^	0, 6, 12 mths
**Secondary outcomes**		
Lower limb joint range of motion (clinician-assessed)	CPIP protocol^28^	0, 6 mths
Motor function (clinician-assessed)	TUG test^29^	0, 6 mths
Independence	GOAL subdomain A^27^	0, 6, 12 mths
Balance	GOAL subdomains A,B,D^27^	0, 6, 12 mths
Pain and discomfort	GOAL subdomain C^27^	0, 6, 12 mths
Health-related quality of life	EQ-5D-Y^30^	0, 6, 12 mths
Educational outcomes	Educational attendance record (days)	0, 6, 12 mths
Exercise adherence	Participant/parent self-reported adherence	6, 12 mths
Additional physiotherapy treatment	Participant/parent self-reported treatment	6, 12 mths

CPIP, Cerebral Palsy Integrated Pathway; EQ-5D-Y, Youth version of EuroQol five-dimension questionnaire; GOAL, Gait Outcomes Assessment List; TUG, Timed Up and Go.

**Table II.** Participant timeline.

Timepoint (from randomization)	Pre-randomization	Baseline	0 to 4 mths	6-mth follow-up	12-mth follow-up
**Enrolment**					
Screening log	✓				
Eligibility confirmed	✓				
Informed consent	✓				
Randomization		✓			
**Interventions**					
Dynamic stretching exercise programme (if randomized to)			✓*		
NHS usual care (if randomized to)			✓*		
**Assessments**					
Baseline demographic questionnaire	✓				
Clinician-assessed outcomes (joint range of motion and motor function)	✓*			✓*	
Participant-assessed outcomes (questionnaire)	✓			✓	✓
Follow-up reminders				✓	✓

* Timepoints that require clinic/hospital attendance, but other assessments at this timepoints could be undertaken electronically/over the telephone.

### Randomization

Consented participants will be randomized (one:one) to either the intervention or control group using the REDCap randomization system (a centralized validated computer randomization programme).^33,34^ Randomization will be performed using a minimization algorithm (including a random element) to ensure balance between the two groups using the following minimization factors: centre, age (four to seven years and eight to 11 years), sex, anatomical CP distribution (bilateral compared with unilateral), and GMFCS levels I and II compared with III.

### Blinding

Participants will be informed of their treatment group by the research facilitator at the initial appointment. Participants will not be blinded to the treatment allocation, nor will the physiotherapists delivering the intervention. The trial statistician and data entry personnel will also not be blinded to the treatment allocation. Physiotherapists performing the six-month follow-up outcome assessment will be blinded to treatment allocation, where possible, provided that staffing levels allow, as will the remaining members of the central trial management team until after the data analysis is complete.

### Interventions

Full details of the SPELL trial interventions and their development will be published elsewhere in accordance with the TIDieR statement;^35^ which are described here in brief:

Dynamic stretching exercise programme: the participants randomized to the dynamic stretching exercise programme will receive a 16-week individually tailored, structured exercise and advice programme, overseen by a physiotherapist, which consists of six one-to-one sessions. The 16-week training period allows time for the neurophysiological response to dynamic stretching and for regular performance of exercises to become part of daily routine.^36^

The six physiotherapy-supervised sessions with the child and the provision of parent/guardian training aim to initiate engagement in longer-term independent exercise. The first physiotherapy session will be up to 90 minutes, followed by five additional physiotherapy sessions of up to 60 minutes. Sessions will be offered at times that minimize disruption to education, consistent with NHS care for this patient group. Appointments will be coordinated so that participants typically start their first exercise session within two to four weeks of randomization, as per local appointment availability. Sessions will be in an outpatient setting or in the participants’ home or educational setting according to clinical need and local service provision. Participants will be advised to complete the exercise programme at home four to five times per week for up to 30 minutes.

The programme allows the participant and family, and physiotherapist, to jointly choose up to five exercise options based on the functional goals identified during the assessment. These will also be based on the participant functional mobility level (GMFCS I to II and III), while ensuring the exercise progression principles are consistent and monitored carefully. The development of the SPELL library of dynamic stretching exercises will be published elsewhere. The library provides a variety of exercises, accommodating different motor function levels and impairments, to allow for an individually tailored programme by selecting those most appropriate to a participant. The library is structured by grouping the exercises under the main target muscle group and allows for progression, ideally within the same muscle groups throughout the programme. The number and type of exercises will be recorded, using treatment logs by the trial physiotherapists. The volume of physiotherapy supervision is broadly consistent with routine practice among children with CP and existing NHS commissioning paradigms.^26^ Importantly, the intervention has been designed to ensure deliverability within an NHS setting.

To support adherence to the dynamic stretching exercise programme, participants will have access to written instructions on the exercises chosen, including photos of each exercise and video instructions of the dynamic stretching exercises chosen via the SPELL study Intervention website.^37^ To ensure accessibility, if families do not have ready access to a mobile device at home, then a device will be loaned to participants to enable them to use the digital intervention app during the supervised exercise period. The intervention design and long-term behaviour change implementation have been underpinned by the capability-opportunity-motivation model of behaviour (COM-B) change for intervention development.^25^ The programme includes goal-setting and exercise diaries accessed via the intervention website, with joint problem-solving, monitoring, and motivation from the physiotherapist. The goal setting and exercise diaries are for use between the participant and their physiotherapist and will be reviewed at each physiotherapy session.

Usual NHS care: children randomized to usual care will attend for a single session with a physiotherapist for an assessment, lasting up to 90 minutes. This will be in addition to physiotherapy appointments that they receive through their routine clinical care. Appointments will be coordinated so that participants typically receive their assessment session within two to four weeks of randomization, as per local appointment availability. To avoid contamination, where possible, physiotherapists delivering usual care will be different to those delivering the dynamic stretching exercise programme. Participants and their parent/carer will be provided with NHS advice on self-management, including a participant information booklet on exercise and activity for children with CP and continuation of any usual fitness/physical activity programme (as applicable).^26^

Participants allocated to the usual care group will not have access to the dynamic stretching exercise programme. Usual care will be recorded using a treatment log maintained on the REDCap trial website by the trial physiotherapists. A guideline on what is considered ‘usual NHS care’ will be provided to the physiotherapists delivering it, and they will be trained to understand the components of this to ensure they know the boundary of provision.

Concomitant care: all participants will be advised they should maintain their usual physiotherapy care, which may include use of orthotics and other forms of treatment during the trial (as long as this does not include a dynamic stretching exercise programme), but will be informed they should use usual clinical referral routes to do so. We will record and monitor any additional physiotherapy received outside of the trial intervention and prescribed during the trial follow-up period.

Training and monitoring of intervention delivery, adherence, and fidelity: physiotherapists delivering the trial interventions will have access to a comprehensive intervention manual and will be required to have undertaken trial-specific training, delivered by a research physiotherapist. The physiotherapists will be experienced practitioners, under the supervision of one of the research physiotherapists on the central trial team. The training will include comprehensive guidance on the theory and practical delivery of the trial interventions.

A rigorous quality control programme will be conducted to ensure protocol and intervention fidelity (i.e. the exercises being undertaken according to the protocol). Quality assurance checks will be made by the trial team, who will observe treatment sessions by physiotherapists. Site visits will be conducted periodically (minimum one visit per site per year) to observe the recruitment, consent, randomization procedures, data collection, follow-up assessments, intervention, and usual care session(s).

We will also monitor adherence to treatment (participants undertaking the prescribed number of sessions and exercises), by logging aspects of the intervention. This will include the name of the exercises prescribed, the duration of physiotherapy appointments attended (and any additional contact), the number of sessions per week undertaken at home without physiotherapy supervision, and whether the session was completed, partially completed, or not completed. Treatment logs will be maintained on the REDCap trial website by the trial physiotherapists. At six and 12 months of follow-up, we will also record longer-term self-reported adherence.

### Outcome measures

The primary outcome is functional mobility at six months measured using the patient-/parent-reported GOAL questionnaire. The GOAL is validated specifically for use in ambulant CP and is internationally accepted as the appropriate functional outcome measure for lower limb interventions in this population. The GOAL questionnaire consists of 48 items grouped into seven domains: A: activities of daily living and independence; B: gait function and mobility; C: pain, discomfort, and fatigue; D: physical activities, sports, and recreation; E: gait pattern and appearance; F: use of braces and mobility aids; and G: body image and self-esteem. A total GOAL score will be calculated in line with the scoring manual, ranging from 0 to 100, with higher values indicating better outcomes.

We will use the child version of the GOAL whenever possible and the parent version one if not. The families will be asked to decide which version is most appropriate as part of the consent process and their decision will be recorded on the baseline clinical assessment form to enable consistent use of the same version during follow-up.

Secondary outcomes (Table I) are:

- Patient-/parent-reported outcomes at six and 12 months: independence measured using the GOAL subdomain A; balance measured using the GOAL subdomains A, B, D; pain and discomfort measured using the GOAL subdomain C;^27^ health-quality of life measured using the EQ-5D-Y;^30^ educational attendance record (number of days off school); exercise adherence; and additional physiotherapy treatment received outside of the trial.
- Clinician assessed outcomes at six months: lower limb joint ROM using the CPIP protocol;^28^ motor function using the Timed up and Go (TUG) test.^29^

The participants’ timeline through the trial is presented in Table II. A flowchart is presented in Figure 1.

### Patient and public involvement

Young people and their families have been involved in the development of this trial in a number of ways including the format of the intervention and choice of primary outcome, and are representatives on the SPELL trial management group and trial steering group.

We have also set up young person/parent advisory groups (YP/PAG) to further support the trial and advise on the trial intervention materials, including the trial website, SPELL intervention app, participant and parent information sheets, and potential strategies to enhance recruitment and retention. In accordance with the Generation R advice (a network of young people supporting design of paediatric research in the UK),^38^ we will work with young people and parents on optimal ways to communicate the results of the trial to patients and the wider public.

### Adverse events

The potential occurrence of adverse events (AEs) related to the intervention (Supplementary Table i) will be recorded. The intervention has been designed to introduce a gradual increase in dynamic ROM, thus minimizing the risk of musculoskeletal injury. Participants and their parent/guardian will be provided with information on the potential AEs resulting from exercise as part of their treatment, including what they should do if they experience an AE, as would happen as part of standard NHS procedures. The participants and their parent/guardian will be asked to notify the treating therapist, as would occur during normal practice, if they suspect that they are suffering an adverse effect. In addition, at the six-month clinical follow-up visit, participants and their parent/guardian will be asked if they have experienced any AEs. Expected general side effects of any form of exercise, such as delayed onset muscle soreness and temporary increases in pain (< seven days), will not be recorded as AEs. Serious adverse events (SAEs) – defined as any unexpected medical occurrence that results in death, is life-threatening, requires hospitalization or prolongation of existing hospitalization, results in persistent or significant disability or incapacity, is otherwise considered medically significant by the investigator – are very rare and are highly unlikely to occur as a result of the intervention delivered in this trial. However, if a SAE arises from the participants’ enrolment on the trial to their allocated intervention, standard procedures for recording and reporting SAEs will then apply.

### Follow-up data collection

Detail of the outcomes to be assessed, how they will be measured, and at which timepoints are shown in Table I. Patient-reported outcomes will be assessed using an electronic (online) questionnaire at six and 12 months from initial randomization. If requested, a paper-based version of the electronic questionnaire will be provided. For those who do not respond to the initial questionnaire, a reminder will be sent two weeks later. Telephone and email follow-up will be used, as applicable, to contact participants, and/or their parent/guardian who do not respond to either the initial or reminder questionnaire. Clinician-assessed outcomes will be assessed at a face-to-face clinic appointment at six months by a blinded physiotherapist, where possible. Participants who do not attend this face-to-face clinic appointment will be contacted by phone by the local site team and a new clinic appointment sent.

### Data management

All data will be processed according to the General Data Protection Regulation (GDPR) and Data Protection Act 2018,^39^ and all documents will be stored safely in confidential conditions. All electronic patient-identifiable information will be held on a secure, password-protected database accessible only to authorized personnel. Paper forms with patient-identifiable information will be held in secure, locked filing cabinets within a restricted area. The processing of the personal data of participants will be minimized wherever possible by the use of a unique participant trial number on trial documents and any electronic databases. Access to participants’ personal identifiable data will be restricted to individuals authorized to have access where this is necessary for their role.

### Sample size

The target sample size for the trial is 334 randomized participants (167 in each treatment group) (Power Analysis and Sample Size (PASS) v. 13).^40^ This will allow detection of a clinically meaningful moderate standardized effect size of 0.4 with a two-sided 5% significance level, 90% power, and allowing for 20% loss to follow-up. The standardized effect size of 0.4 corresponds to a difference of 6.8 points on the GOAL outcome measure,^27^ which ranges from 0 to 100, with a SD of 17. A difference of 6.8 is considered functionally important and achievable by key stakeholders, including patients who provided input in focus groups, and clinicians we surveyed in preparation for the trial. SDs of this magnitude have been reported in similar patient populations.^27,41^ The sample size assumptions will be reviewed after approximately 50% of the participants have been recruited.

### Statistical analysis

The primary analysis will use the randomized (intention-to-treat (ITT)) population, analyzing participants with available outcome data in their randomized groups, regardless of adherence to their allocated intervention. Primary and secondary outcome analyses will use two-sided 5% significance and 95% CIs with associated p-values.

Differences in GOAL scores between the trial groups will be estimated using a multilevel mixed effects regression model, allowing for repeated measures clustered within participants and for potential clustering within randomizing sites. The model will be adjusted for minimization factors (sex, anatomical disease distribution (bilateral compared with unilateral CP), and GMFCS level (levels I and II compared with III)) and other important prognostic factors (i.e. neurological pattern, epilepsy, or visual impairment), including the baseline GOAL scores. A treatment by time interaction will be included, indicating the protocol stipulated follow-up time to which the assessment refers. Model diagnostics, including approximate normality of the residuals, will be assessed. Adjusted mean differences and unadjusted mean differences between groups will be presented together with 95% CIs.

We will explore the effect of non-adherence with the randomized interventions using complier-average causal effect (CACE) analyses. Adherence will be defined as having completed all six physiotherapy sessions. However, the treating physiotherapist may decide that treatment is completed over a smaller number of sessions, depending on the progress of the child. Secondary outcomes will be analyzed using generalized linear models, with model adjustment as described for the primary analysis above. Subgroup analysis using predefined subgroups will investigate potential outcome predictors such as sex, anatomical distribution of the condition, GMFCS level, or other baseline characteristics. A statistical analysis plan (SAP) with full details of all analyses planned for the study data will be drafted early in the trial and finalized prior to any primary outcome analysis.

### Missing data

Missing data will be reported and summarized by group. The distribution of missing primary outcome data will be explored to assess the assumption of data missing at random. The potential impact of informative missing data (missing not at random) on the treatment effect will be investigated.

## Ethics and dissemination

Ethical approval was obtained from the East of England – Essex Research Ethics Committee (REC: 23/EE/0153) and prospectively registered (ISRCTN 15808719). A data safety and monitoring committee has been appointed to independently review data on safety, protocol adherence, and recruitment in accordance with the Data Monitoring Committees: Lessons, Ethics, Statistics (DAMOCLES) charter.^42^ Direct access to research data will be granted to authorized representatives of the sponsor (University of Oxford, UK), regulatory authorities, or the host institution for monitoring and/or auditing of the trial to ensure compliance with regulations. Summary results will be included on the ISRCTN database within six months of the end of the trial. Requests for data (anonymized trial participant level data) will be provided at the end of the trial to external researchers who provide a methodologically sound proposal to the trial team (and who will be required to sign a data sharing access agreement with the sponsor), and in accordance with the National Institute for Health and Care Research (NIHR) guidance.

Trial findings will inform clinical practice for the management of ambulant children with spastic CP and its results will be published in a high-impact open-access journal, in accordance with the NIHR’s policy on open access research. The trial results will be reported following the CONSORT guideline.^43^ We will use the TIDieR statement for reporting the intervention, ensuring that replication is possible. Trial materials, including the physiotherapist training materials and intervention materials, will be made freely available via the trial website. Participants and their parent/guardian will be asked if and how they would like to be informed of the results as part of the consent process.

**Take home message**

- Dynamic stretching exercises are one of the interventions frequently prescribed by physiotherapists for children with cerebral palsy (CP).

- However, there is wide variability in the exercise regimes used and limited evidence of their effectiveness.

- This article describes the protocol for the SPELL trial, which is a mutlicentre trial, looking at the clinical effectiveness of an individually tailored dynamic stretching programme compared to usual care for ambulant children with spastic CP.

## Data Availability

The data that support the findings for this study are available to other researchers from the corresponding author upon reasonable request.

## References

[1] Rosenbaum P Paneth N Leviton A et al. A report: the definition and classification of cerebral palsy April 2006 Dev Med Child Neurol Suppl 2007 109 8 14 17370477

[2] No authors listed Cerebral palsy: introduction 2020 SCOPE https://www.scope.org.uk/advice-and-support/cerebral-palsy-introduction date last accessed 10 April 2025

[3] Palisano RJ Rosenbaum P Bartlett D Livingston MH Content validity of the expanded and revised gross motor function classification system Dev Med Child Neurol 2008 50 10 744 750 10.1111/j.1469-8749.2008.03089.x 18834387

[4] Lieber RL Theologis T Muscle-tendon unit in children with cerebral palsy Dev Med Child Neurol 2021 63 8 908 913 10.1111/dmcn.14807 33426691

[5] Ostojic K Paget S Kyriagis M Morrow A Acute and chronic pain in children and adolescents with cerebral palsy: prevalence, interference, and management Arch Phys Med Rehabil 2020 101 2 213 219 10.1016/j.apmr.2019.08.475 31521713

[6] Ryan JM Lavelle G Theis N Kilbride C Noorkoiv M Patterns of health service use among young people with cerebral palsy in England Front Neurol 2021 12 659031 10.3389/fneur.2021.659031 34054701 PMC8153484

[7] Morris C Simkiss D Busk M et al. Setting research priorities to improve the health of children and young people with neurodisability: a British Academy of Childhood Disability-James Lind Alliance Research Priority Setting Partnership BMJ Open 2015 5 1 e006233 10.1136/bmjopen-2014-006233 25631309 PMC4316435

[8] Vella-Baldacchino M Perry DC Roposch A et al. Research priorities in children requiring elective surgery for conditions affecting the lower limbs: a James Lind Alliance Priority Setting Partnership BMJ Open 2019 9 12 e033233 10.1136/bmjopen-2019-033233 31892663 PMC6955494

[9] Beresford B Clarke S Maddison J Therapy interventions for children with neurodisabilities: a qualitative scoping study Health Technol Assess 2018 22 3 1 150 10.3310/hta22030 29345224 PMC5787698

[10] Graham HK Thomason P Willoughby K et al. Musculoskeletal pathology in cerebral palsy: a classification system and reliability study Children (Basel) 2021 8 3 252 10.3390/children8030252 33807084 PMC8004848

[11] Eldridge F Lavin N How effective is stretching in maintaining range of movement for children with cerebral palsy? A critical review Int J Ther Rehabil 2016 23 8 386 395 10.12968/ijtr.2016.23.8.386

[12] Walhain F Desloovere K Declerck M Van Campenhout A Bar-On L Interventions and lower-limb macroscopic muscle morphology in children with spastic cerebral palsy: a scoping review Dev Med Child Neurol 2021 63 3 274 286 10.1111/dmcn.14652 32876960

[13] Taflampas G Kilbride C Levin W Lavelle G Ryan JM Interventions to improve or maintain lower-limb function among ambulatory adolescents with cerebral palsy: a cross-sectional survey of current practice in the UK Phys Occup Ther Pediatr 2018 38 4 355 369 10.1080/01942638.2017.1400490 29220616

[14] Verschuren O Peterson MD Balemans ACJ Hurvitz EA Exercise and physical activity recommendations for people with cerebral palsy Dev Med Child Neurol 2016 58 8 798 808 10.1111/dmcn.13053 26853808 PMC4942358

[15] Opplert J Babault N Acute effects of dynamic stretching on muscle flexibility and performance: an analysis of the current literature Sports Med 2018 48 2 299 325 10.1007/s40279-017-0797-9 29063454

[16] Iwata M Yamamoto A Matsuo S et al. Dynamic stretching has sustained effects on range of motion and passive stiffness of the hamstring muscles J Sports Sci Med 2019 18 1 13 20 30787647 PMC6370952

[17] Hösl M Böhm H Eck J Döderlein L Arampatzis A Effects of backward-downhill treadmill training versus manual static plantarflexor stretching on muscle-joint pathology and function in children with spastic cerebral palsy Gait Posture 2018 65 121 128 10.1016/j.gaitpost.2018.07.171 30558918

[18] Lee SJ Jin D Kang SH Gaebler-Spira D Zhang LQ Combined ankle/knee stretching and pivoting stepping training for children with cerebral palsy IEEE Trans Neural Syst Rehabil Eng 2019 27 9 1743 1752 10.1109/TNSRE.2019.2934139 31403432 PMC6779478

[19] Fauzi AA Khayat MM Sabirin S Haron N Mohamed MNA Davis GM Structured home-based exercise program for improving walking ability in ambulant children with cerebral palsy J Pediatr Rehabil Med 2019 12 2 161 169 10.3233/PRM-180538 31227664

[20] Fosdahl MA Jahnsen R Kvalheim K Holm I Effect of a combined stretching and strength training program on gait function in children with cerebral palsy, GMFCS Level I & II: a randomized controlled trial Medicina (Kaunas) 2019 55 6 250 10.3390/medicina55060250 31174397 PMC6630432

[21] Kalkman BM Bar-On L O’Brien TD Maganaris CN Stretching interventions in children with cerebral palsy: why are they ineffective in improving muscle function and how can we better their outcome? Front Physiol 2020 11 131 10.3389/fphys.2020.00131 32153428 PMC7047287

[22] Kimoto M Okada K Sakamoto H Kondou T Kawanobe U Relationship between walking efficiency and muscular strength of the lower limbs in children with cerebral palsy J Phys Ther Sci 2019 31 3 232 235 10.1589/jpts.31.232 30936637 PMC6428656

[23] Coman C Meldrum D Kiernan D Malone A Pilates-based exercises for gait and balance in ambulant children with cerebral palsy: feasibility and clinical outcomes of a randomised controlled trial Disabil Rehabil 2023 45 17 2796 2807 10.1080/09638288.2022.2110617 35996891

[24] Meade LB Bearne LM Sweeney LH Alageel SH Godfrey EL Behaviour change techniques associated with adherence to prescribed exercise in patients with persistent musculoskeletal pain: systematic review Br J Health Psychol 2019 24 1 10 30 10.1111/bjhp.12324 29911311 PMC6585717

[25] Michie S van Stralen MM West R The behaviour change wheel: a new method for characterising and designing behaviour change interventions Implement Sci 2011 6 42 10.1186/1748-5908-6-42 21513547 PMC3096582

[26] No authors listed Clinical guideline [CG145]. Spasticity in under 19s: management National Institute for Health and Care Excellence (NICE https://www.nice.org.uk/guidance/cg145 date last accessed 10 April 2025

[27] Thomason P Tan A Donnan A Rodda J Graham HK Narayanan U The Gait Outcomes Assessment List (GOAL): validation of a new assessment of gait function for children with cerebral palsy Dev Med Child Neurol 2018 60 6 618 623 10.1111/dmcn.13722 29573409

[28] No authors listed CPIP-UK National Network The Association of Paediatric Chartered Physiotherapists https://apcp.csp.org.uk/content/cpip-uk-national-network date last accessed 10 April 2025

[29] Carey H Martin K Combs-Miller S Heathcock JC Reliability and responsiveness of the timed up and go test in children with cerebral palsy Pediatr Phys Ther 2016 28 4 401 408 10.1097/PEP.0000000000000301 27661230

[30] Wille N Badia X Bonsel G Burström K Cavrini G Devlin N et al. Development of the EQ-5D-Y: a child-friendly version of the EQ-5D. Quality of life research: an international journal of quality of life aspects of treatment, care and rehabilitation 2010 19 6 875 886 10.1007/s11136-010-9648-y 20405245 PMC2892611

[31] HopewellS KeeneDJ MarianIR et al. Clinical effectiveness of an individually tailored strengthening programme, including progressive resistance exercises and advice, compared to usual care for ambulant adolescents with spastic cerebral palsy (ROBUST trial): a parallel group randomized controlled trial Bone Jt Open 2025 6 5 10.1302/2633-1462.65.BJO-2024-026840306695

[32] No authors listed Research involving children NHS Health Research Authority 2024 https://www.hra.nhs.uk/planning-and-improving-research/policies-standards-legislation/research-involving-children/ date last accessed 10 April 2025

[33] Harris PA Taylor R Thielke R Payne J Gonzalez N Conde JG Research electronic data capture (REDCap)--a metadata-driven methodology and workflow process for providing translational research informatics support J Biomed Inform 2009 42 2 377 381 10.1016/j.jbi.2008.08.010 18929686 PMC2700030

[34] Harris PA Taylor R Minor BL et al. The REDCap consortium: Building an international community of software platform partners J Biomed Inform 2019 95 103208 10.1016/j.jbi.2019.103208 31078660 PMC7254481

[35] Hoffmann TC Glasziou PP Boutron I et al. Better reporting of interventions: template for intervention description and replication (TIDieR) checklist and guide BMJ 2014 348 mar07 3 g1687 10.1136/bmj.g1687 24609605

[36] Lally P van Jaarsveld CHM Potts HWW Wardle J How are habits formed: Modelling habit formation in the real world Euro J Social Psych 2010 40 6 998 1009 10.1002/ejsp.674

[37] No authors listed The SPELL Study https://spell-study.org date last accessed 10 April 2025

[38] No authors listed Generation R: young people improving research https://generationr.org.uk/ date last accessed 15 April 2025

[39] No authors listed Data protection UK Government https://www.gov.uk/data-protection date last accessed 10 April 2025

[40] No authors listed PASS Statistical, Graphics, and Sample Size Software NCSS Statistical Analysis www.ncss.com date last accessed 10 April 2025

[41] Thomason P Graham HK The gait outcomes assessment list (GOAL): responsiveness to change in gait function for children with cerebral palsy Develop Med Child Neuro 2017 59 S3 85 86 10.1111/dmcn.11_13512 29573409

[42] DAMOCLES Study Group, NHS Health Technology Assessment Programme A proposed charter for clinical trial data monitoring committees: helping them to do their job well Lancet 2005 365 9460 711 722 10.1016/S0140-6736(05)17965-3 15721478

[43] No authors listed CONSORT 2010 Statement: updated guidelines for reporting parallel group randomised trials https://www.equator-network.org/reporting-guidelines/consort/ date last accessed 10 April 2025 10.1016/j.ijsu.2011.09.004

